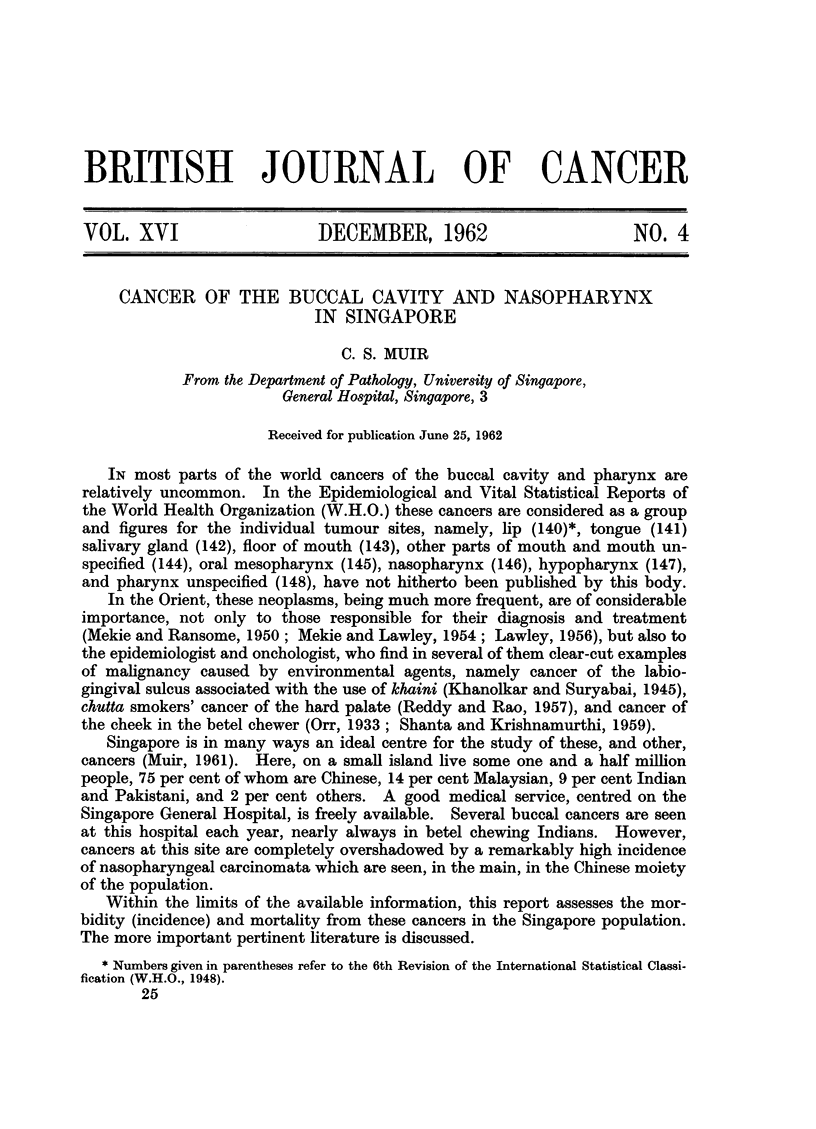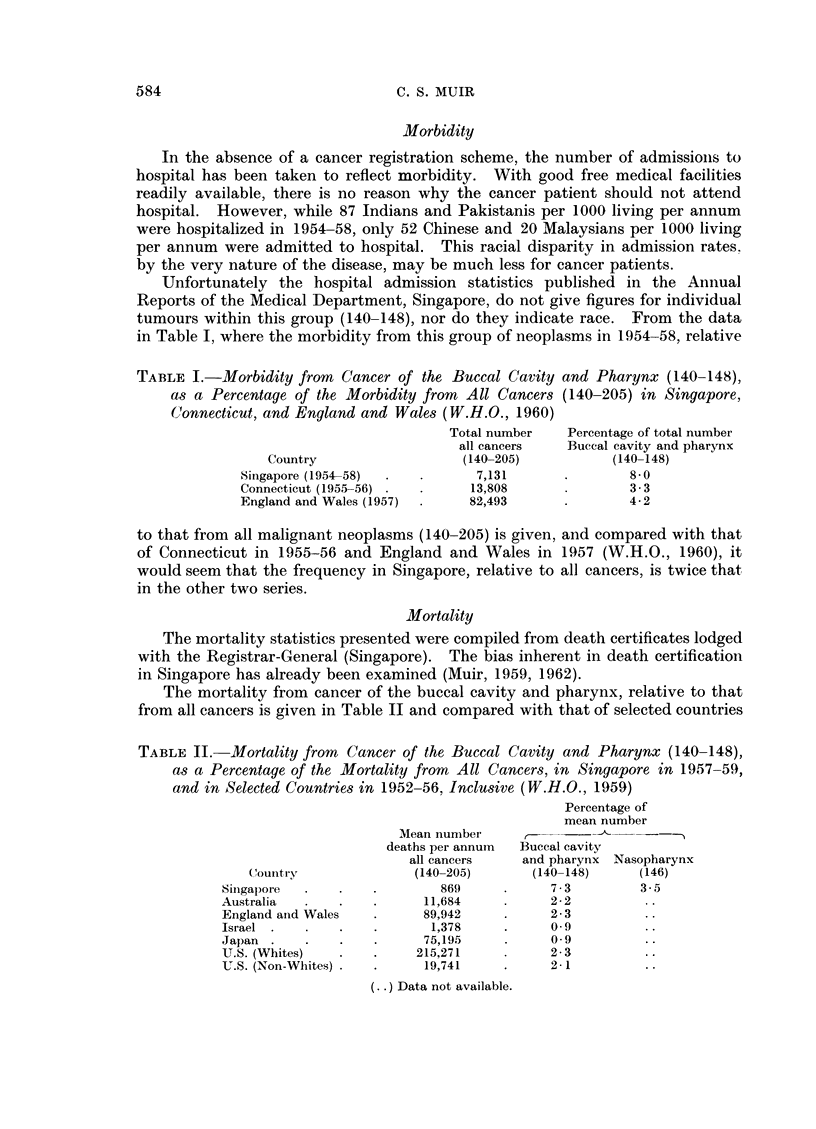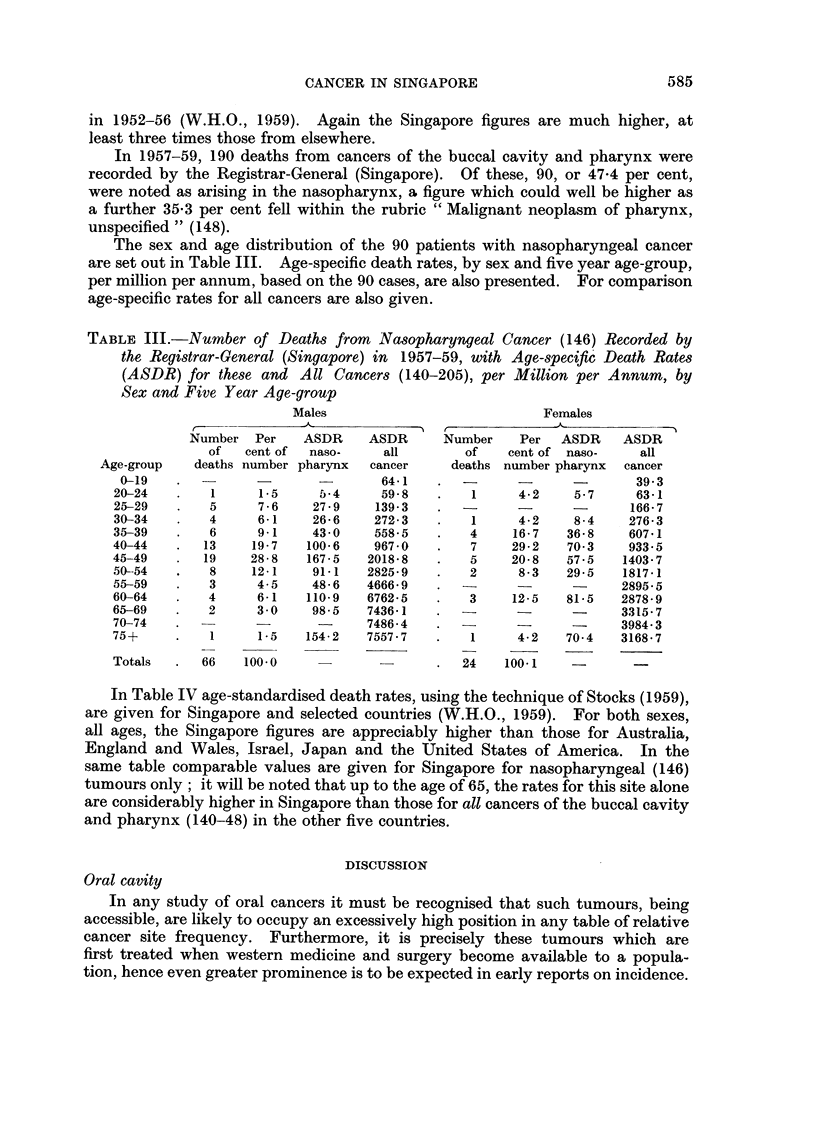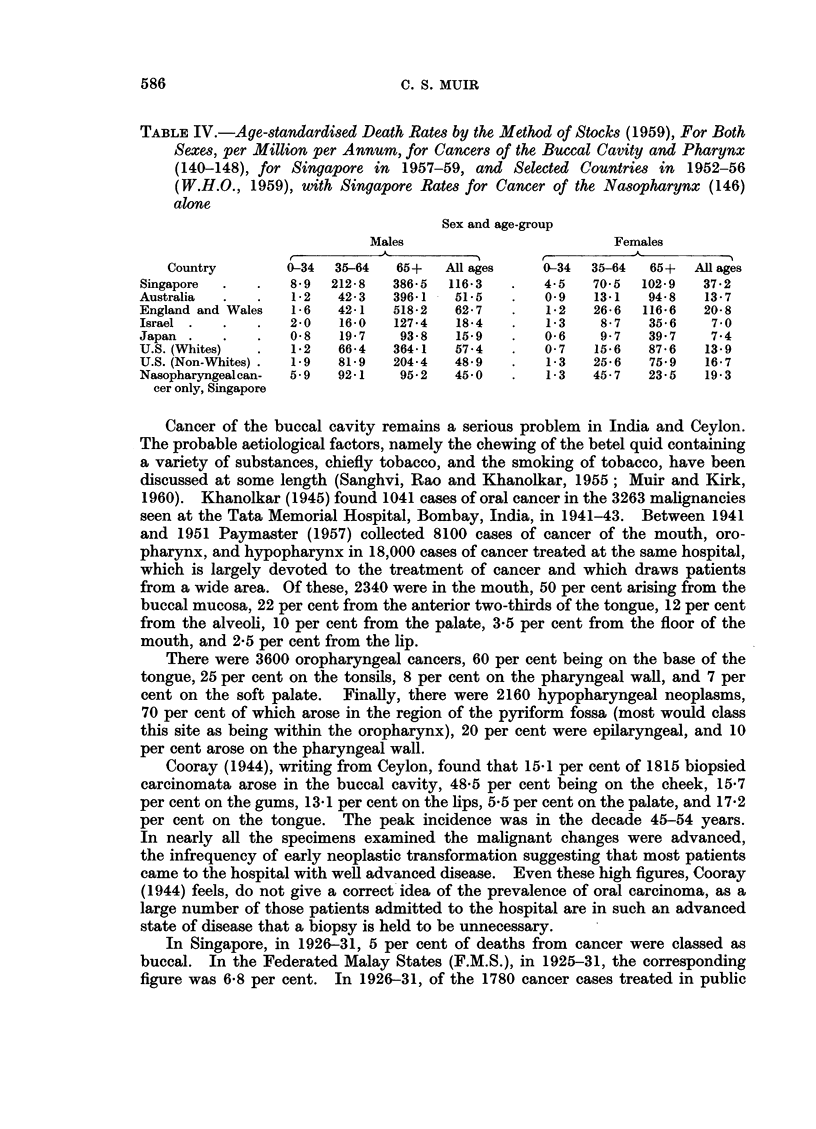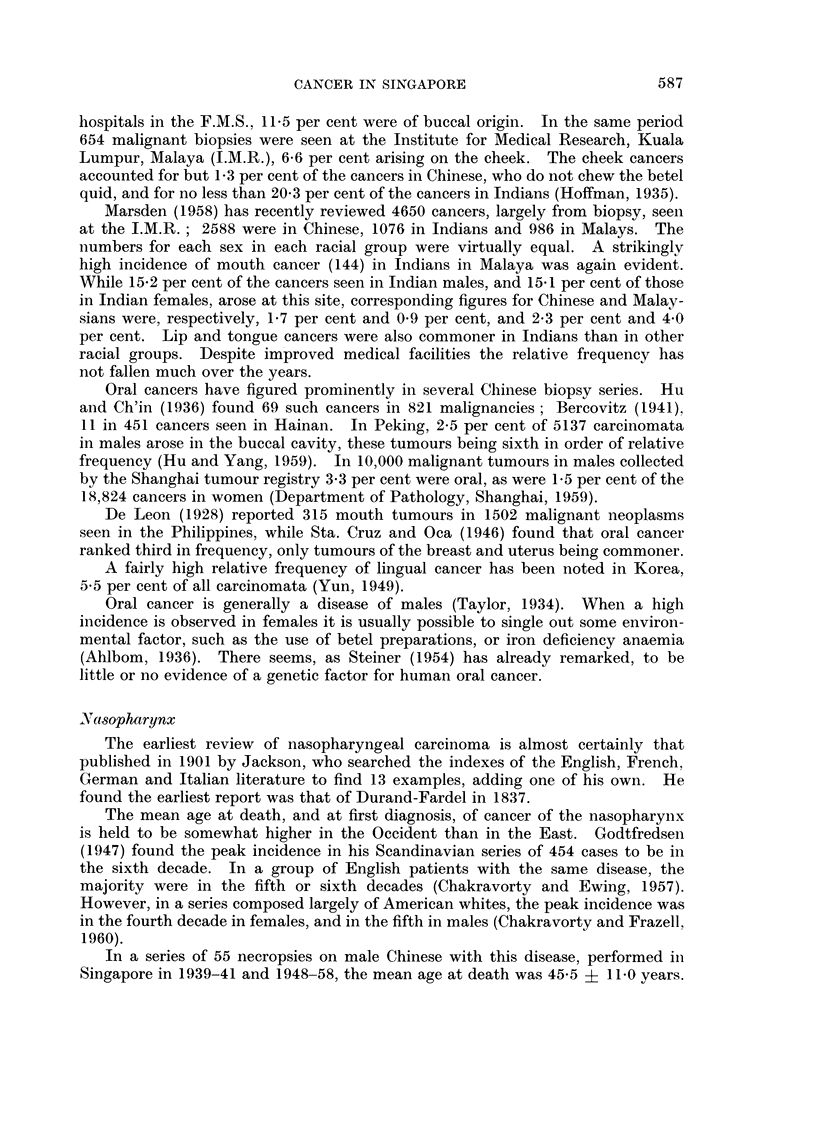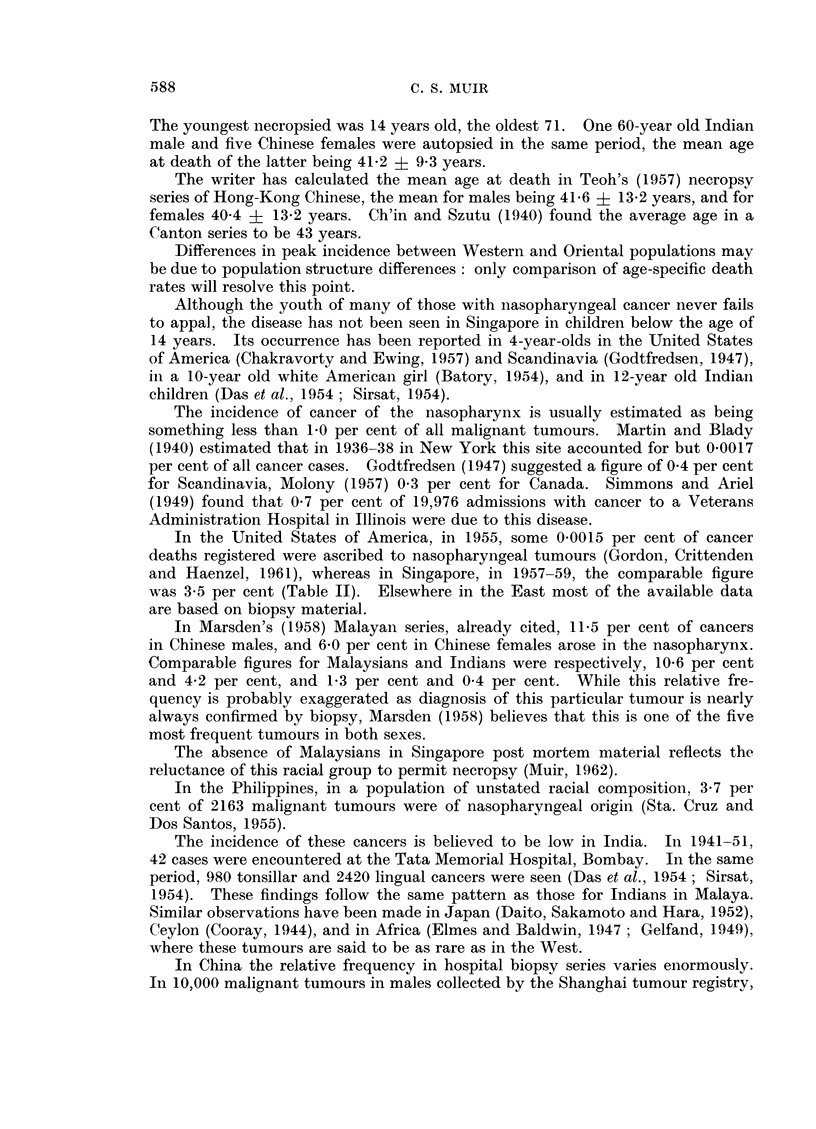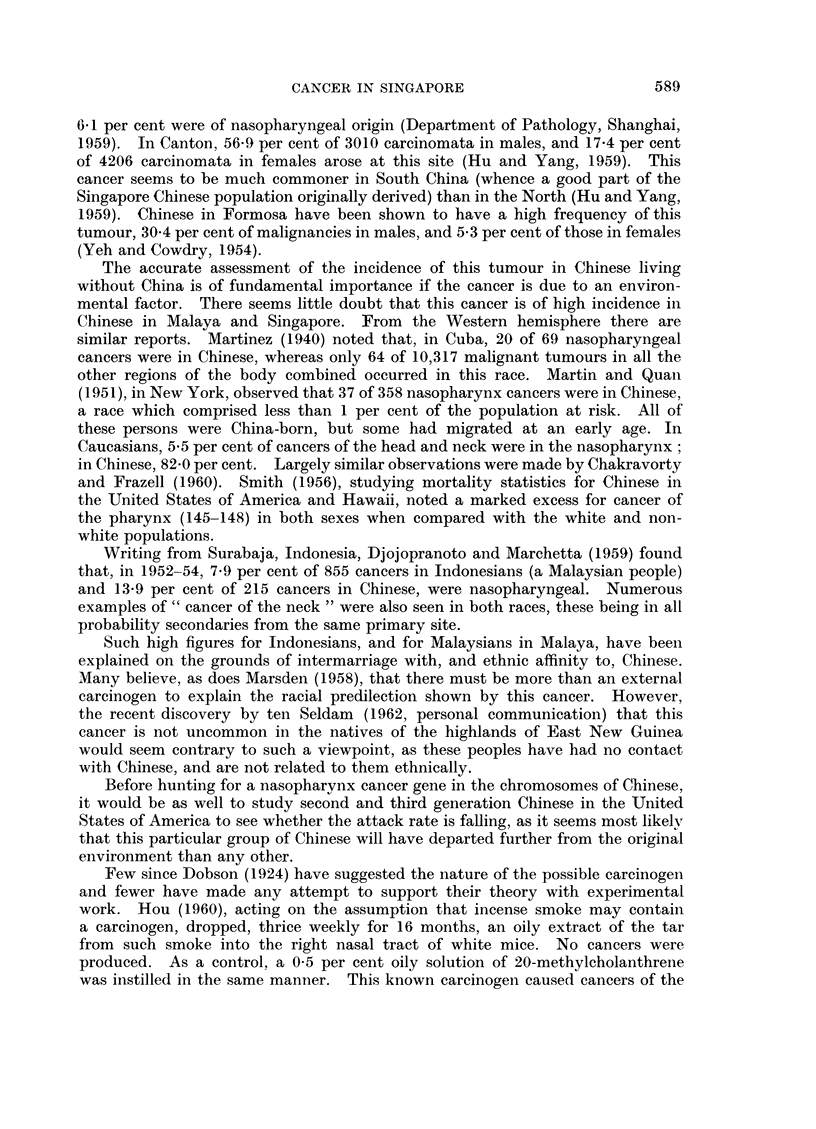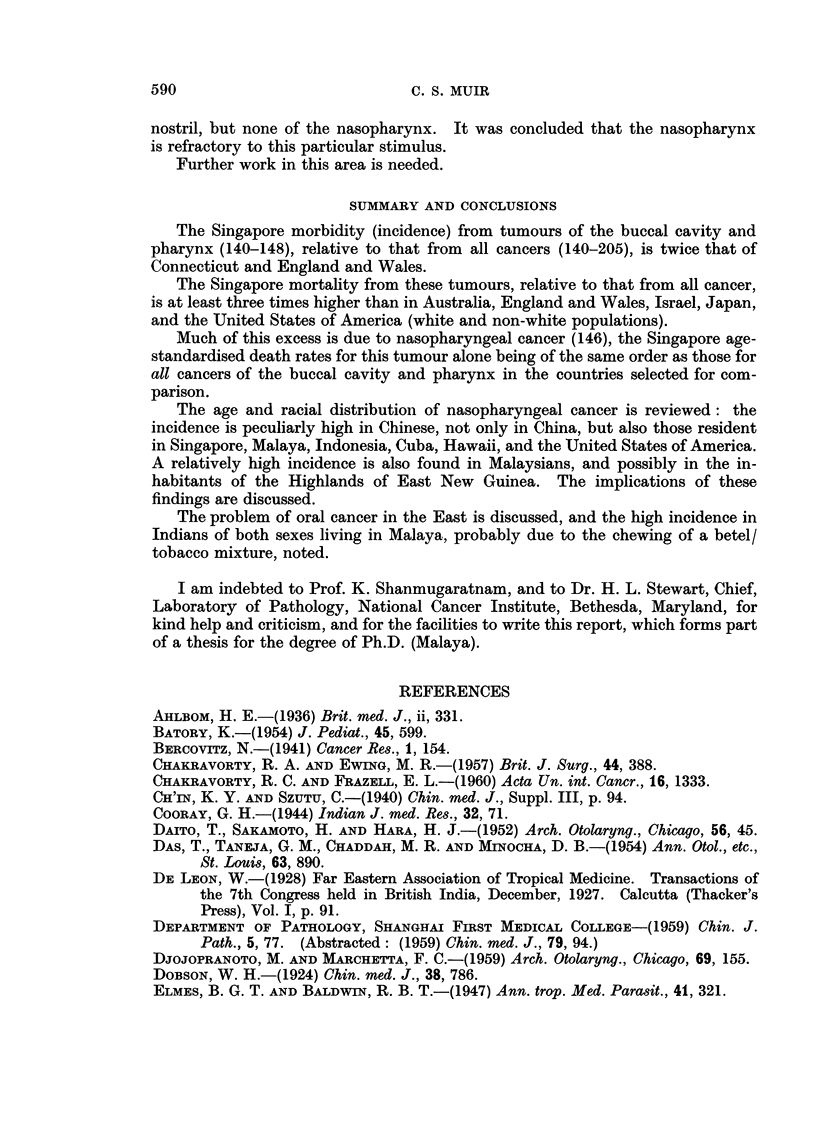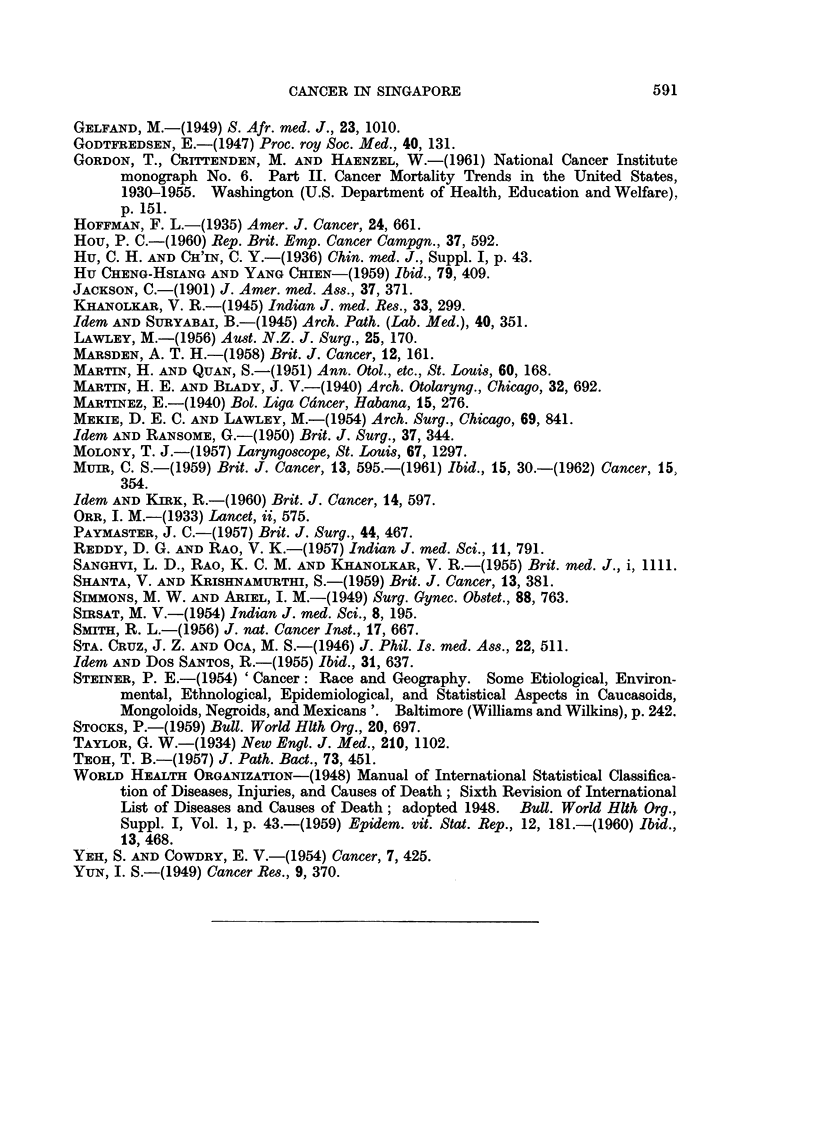# Cancer of the Buccal Cavity and Nasopharynx in Singapore

**DOI:** 10.1038/bjc.1962.68

**Published:** 1962-12

**Authors:** C. S. Muir


### BRITISH JOURNAL OF CANCER

VOL. XVI DECEMBER, 1962 NO. 4

### CANCER OF THE BUCCAL CAVITY AND NASOPHARYNX IN SINGAPORE

C. S. MUIR

*From the Department of Pathology, University of Singapore, General Hospital, Singapore, 3*

Received for publication June 25, 1962

IN most parts of the world cancers of the buccal cavity and pharynx are relatively uncommon. In the Epidemiological and Vital Statistical Reports of the World Health Organization (W.H.O.) these cancers are considered as a group and figures for the individual tumour sites, namely, lip (140)*, tongue (141) salivary gland (142), floor of mouth (143), other parts of mouth and mouth unspecified (144), oral mesopharynx (145), nasopharynx (146), hypopharynx (147), and pharynx unspecified (148), have not hitherto been published by this body.

In the Orient, these neoplasms, being much more frequent, are of considerable importance, not only to those responsible for their diagnosis and treatment (Mekie and Ransome, 1950 ; Mekie and Lawley, 1954 ; Lawley, 1956), but also to the epidemiologist and onchologist, who find in several of them clear-cut examples of malignancy caused by environmental agents, namely cancer of the labio-gingival sulcus associated with the use of *khaini* (Khanolkar and Suryabai, 1945), *chutta* smokers' cancer of the hard palate (Reddy and Rao, 1957), and cancer of the cheek in the betel chewer (Orr, 1933 ; Shanta and Krishnamurthi, 1959).

Singapore is in many ways an ideal centre for the study of these, and other, cancers (Muir, 1961). Here, on a small island live some one and a half million people, 75 per cent of whom are Chinese, 14 per cent Malaysian, 9 per cent Indian and Pakistani, and 2 per cent others. A good medical service, centred on the Singapore General Hospital, is freely available. Several buccal cancers are seen at this hospital each year, nearly always in betel chewing Indians. However, cancers at this site are completely overshadowed by a remarkably high incidence of nasopharyngeal carcinomata which are seen, in the main, in the Chinese moiety of the population.

Within the limits of the available information, this report assesses the morbidity (incidence) and mortality from these cancers in the Singapore population. The more important pertinent literature is discussed.

* Numbers given in parentheses refer to the 6th Revision of the International Statistical Classification (W.H.O., 1948).

### *Morbidity*

In the absence of a cancer registration scheme, the number of admissions to hospital has been taken to reflect morbidity. With good free medical facilities readily available, there is no reason why the cancer patient should not attend hospital. However, while 87 Indians and Pakistanis per 1000 living per annum were hospitalized in 1954–58, only 52 Chinese and 20 Malaysians per 1000 living per annum were admitted to hospital. This racial disparity in admission rates, by the very nature of the disease, may be much less for cancer patients.

Unfortunately the hospital admission statistics published in the Annual Reports of the Medical Department, Singapore, do not give figures for individual tumours within this group (140–148), nor do they indicate race. From the data in Table I, where the morbidity from this group of neoplasms in 1954–58, relative

TABLE I.—*Morbidity from Cancer of the Buccal Cavity and Pharynx* (140–148), *as a Percentage of the Morbidity from All Cancers* (140–205) *in Singapore, Connecticut, and England and Wales* (*W.H.O.*, 1960)

| Country | Total number all cancers (140–205) | Percentage of total number Buccal cavity and pharynx (140–148) |
|---|---|---|
| Singapore (1954–58) | 7,131 | 8·0 |
| Connecticut (1955–56) | 13,808 | 3·3 |
| England and Wales (1957) | 82,493 | 4·2 |

to that from all malignant neoplasms (140–205) is given, and compared with that of Connecticut in 1955–56 and England and Wales in 1957 (W.H.O., 1960), it would seem that the frequency in Singapore, relative to all cancers, is twice that in the other two series.

### *Mortality*

The mortality statistics presented were compiled from death certificates lodged with the Registrar-General (Singapore). The bias inherent in death certification in Singapore has already been examined (Muir, 1959, 1962).

The mortality from cancer of the buccal cavity and pharynx, relative to that from all cancers is given in Table II and compared with that of selected countries

TABLE II.—*Mortality from Cancer of the Buccal Cavity and Pharynx* (140–148), *as a Percentage of the Mortality from All Cancers, in Singapore in* 1957–59, *and in Selected Countries in* 1952–56, *Inclusive* (*W.H.O.*, 1959)

| Country | Mean number deaths per annum all cancers (140–205) | Percentage of mean number: Buccal cavity and pharynx (140–148) | Percentage of mean number: Nasopharynx (146) |
|---|---|---|---|
| Singapore | 869 | 7·3 | 3·5 |
| Australia | 11,684 | 2·2 | . . |
| England and Wales | 89,942 | 2·3 | . . |
| Israel | 1,378 | 0·9 | . . |
| Japan | 75,195 | 0·9 | . . |
| U.S. (Whites) | 215,271 | 2·3 | . . |
| U.S. (Non-Whites) | 19,741 | 2·1 | . . |

(. .) Data not available.

in 1952–56 (W.H.O., 1959). Again the Singapore figures are much higher, at least three times those from elsewhere.

In 1957–59, 190 deaths from cancers of the buccal cavity and pharynx were recorded by the Registrar-General (Singapore). Of these, 90, or 47·4 per cent, were noted as arising in the nasopharynx, a figure which could well be higher as a further 35·3 per cent fell within the rubric " Malignant neoplasm of pharynx, unspecified " (148).

The sex and age distribution of the 90 patients with nasopharyngeal cancer are set out in Table III. Age-specific death rates, by sex and five year age-group, per million per annum, based on the 90 cases, are also presented. For comparison age-specific rates for all cancers are also given.

TABLE III.—*Number of Deaths from Nasopharyngeal Cancer* (146) *Recorded by the Registrar-General* (*Singapore*) *in* 1957–59, *with Age-specific Death Rates* (*ASDR*) *for these and All Cancers* (140–205), *per Million per Annum, by Sex and Five Year Age-group*

| | Males | | | | Females | | | |
|---|---|---|---|---|---|---|---|---|
| Age-group | Number of deaths | Per cent of number | ASDR naso-pharynx | ASDR all cancer | Number of deaths | Per cent of number | ASDR naso-pharynx | ASDR all cancer |
| 0–19 | — | — | — | 64·1 | — | — | — | 39·3 |
| 20–24 | 1 | 1·5 | 5·4 | 59·8 | 1 | 4·2 | 5·7 | 63·1 |
| 25–29 | 5 | 7·6 | 27·9 | 139·3 | — | — | — | 166·7 |
| 30–34 | 4 | 6·1 | 26·6 | 272·3 | 1 | 4·2 | 8·4 | 276·3 |
| 35–39 | 6 | 9·1 | 43·0 | 558·5 | 4 | 16·7 | 36·8 | 607·1 |
| 40–44 | 13 | 19·7 | 100·6 | 967·0 | 7 | 29·2 | 70·3 | 933·5 |
| 45–49 | 19 | 28·8 | 167·5 | 2018·8 | 5 | 20·8 | 57·5 | 1403·7 |
| 50–54 | 8 | 12·1 | 91·1 | 2825·9 | 2 | 8·3 | 29·5 | 1817·1 |
| 55–59 | 3 | 4·5 | 48·6 | 4666·9 | — | — | — | 2895·5 |
| 60–64 | 4 | 6·1 | 110·9 | 6762·5 | 3 | 12·5 | 81·5 | 2878·9 |
| 65–69 | 2 | 3·0 | 98·5 | 7436·1 | — | — | — | 3315·7 |
| 70–74 | — | — | — | 7486·4 | — | — | — | 3984·3 |
| 75+ | 1 | 1·5 | 154·2 | 7557·7 | 1 | 4·2 | 70·4 | 3168·7 |
| Totals | 66 | 100·0 | — | — | 24 | 100·1 | — | — |

In Table IV age-standardised death rates, using the technique of Stocks (1959), are given for Singapore and selected countries (W.H.O., 1959). For both sexes, all ages, the Singapore figures are appreciably higher than those for Australia, England and Wales, Israel, Japan and the United States of America. In the same table comparable values are given for Singapore for nasopharyngeal (146) tumours only ; it will be noted that up to the age of 65, the rates for this site alone are considerably higher in Singapore than those for *all* cancers of the buccal cavity and pharynx (140–48) in the other five countries.

### DISCUSSION

### *Oral cavity*

In any study of oral cancers it must be recognised that such tumours, being accessible, are likely to occupy an excessively high position in any table of relative cancer site frequency. Furthermore, it is precisely these tumours which are first treated when western medicine and surgery become available to a population, hence even greater prominence is to be expected in early reports on incidence.

TABLE IV.—*Age-standardised Death Rates by the Method of Stocks* (1959), *For Both Sexes, per Million per Annum, for Cancers of the Buccal Cavity and Pharynx* (140–148), *for Singapore in* 1957–59, *and Selected Countries in* 1952–56 (*W.H.O.*, 1959), *with Singapore Rates for Cancer of the Nasopharynx* (146) *alone*

| | Sex and age-group | | | | | | | |
|---|---|---|---|---|---|---|---|---|
| | Males | | | | Females | | | |
| Country | 0–34 | 35–64 | 65+ | All ages | 0–34 | 35–64 | 65+ | All ages |
| Singapore | 8·9 | 212·8 | 386·5 | 116·3 | 4·5 | 70·5 | 102·9 | 37·2 |
| Australia | 1·2 | 42·3 | 396·1 | 51·5 | 0·9 | 13·1 | 94·8 | 13·7 |
| England and Wales | 1·6 | 42·1 | 518·2 | 62·7 | 1·2 | 26·6 | 116·6 | 20·8 |
| Israel | 2·0 | 16·0 | 127·4 | 18·4 | 1·3 | 8·7 | 35·6 | 7·0 |
| Japan | 0·8 | 19·7 | 93·8 | 15·9 | 0·6 | 9·7 | 39·7 | 7·4 |
| U.S. (Whites) | 1·2 | 66·4 | 364·1 | 57·4 | 0·7 | 15·6 | 87·6 | 13·9 |
| U.S. (Non-Whites) | 1·9 | 81·9 | 204·4 | 48·9 | 1·3 | 25·6 | 75·9 | 16·7 |
| Nasopharyngeal cancer only, Singapore | 5·9 | 92·1 | 95·2 | 45·0 | 1·3 | 45·7 | 23·5 | 19·3 |

Cancer of the buccal cavity remains a serious problem in India and Ceylon. The probable aetiological factors, namely the chewing of the betel quid containing a variety of substances, chiefly tobacco, and the smoking of tobacco, have been discussed at some length (Sanghvi, Rao and Khanolkar, 1955 ; Muir and Kirk, 1960). Khanolkar (1945) found 1041 cases of oral cancer in the 3263 malignancies seen at the Tata Memorial Hospital, Bombay, India, in 1941–43. Between 1941 and 1951 Paymaster (1957) collected 8100 cases of cancer of the mouth, oropharynx, and hypopharynx in 18,000 cases of cancer treated at the same hospital, which is largely devoted to the treatment of cancer and which draws patients from a wide area. Of these, 2340 were in the mouth, 50 per cent arising from the buccal mucosa, 22 per cent from the anterior two-thirds of the tongue, 12 per cent from the alveoli, 10 per cent from the palate, 3·5 per cent from the floor of the mouth, and 2·5 per cent from the lip.

There were 3600 oropharyngeal cancers, 60 per cent being on the base of the tongue, 25 per cent on the tonsils, 8 per cent on the pharyngeal wall, and 7 per cent on the soft palate. Finally, there were 2160 hypopharyngeal neoplasms, 70 per cent of which arose in the region of the pyriform fossa (most would class this site as being within the oropharynx), 20 per cent were epilaryngeal, and 10 per cent arose on the pharyngeal wall.

Cooray (1944), writing from Ceylon, found that 15·1 per cent of 1815 biopsied carcinomata arose in the buccal cavity, 48·5 per cent being on the cheek, 15·7 per cent on the gums, 13·1 per cent on the lips, 5·5 per cent on the palate, and 17·2 per cent on the tongue. The peak incidence was in the decade 45–54 years. In nearly all the specimens examined the malignant changes were advanced, the infrequency of early neoplastic transformation suggesting that most patients came to the hospital with well advanced disease. Even these high figures, Cooray (1944) feels, do not give a correct idea of the prevalence of oral carcinoma, as a large number of those patients admitted to the hospital are in such an advanced state of disease that a biopsy is held to be unnecessary.

In Singapore, in 1926–31, 5 per cent of deaths from cancer were classed as buccal. In the Federated Malay States (F.M.S.), in 1925–31, the corresponding figure was 6·8 per cent. In 1926–31, of the 1780 cancer cases treated in public hospitals in the F.M.S., 11·5 per cent were of buccal origin. In the same period 654 malignant biopsies were seen at the Institute for Medical Research, Kuala Lumpur, Malaya (I.M.R.), 6·6 per cent arising on the cheek. The cheek cancers accounted for but 1·3 per cent of the cancers in Chinese, who do not chew the betel quid, and for no less than 20·3 per cent of the cancers in Indians (Hoffman, 1935).

Marsden (1958) has recently reviewed 4650 cancers, largely from biopsy, seen at the I.M.R.; 2588 were in Chinese, 1076 in Indians and 986 in Malays. The numbers for each sex in each racial group were virtually equal. A strikingly high incidence of mouth cancer (144) in Indians in Malaya was again evident. While 15·2 per cent of the cancers seen in Indian males, and 15·1 per cent of those in Indian females, arose at this site, corresponding figures for Chinese and Malaysians were, respectively, 1·7 per cent and 0·9 per cent, and 2·3 per cent and 4·0 per cent. Lip and tongue cancers were also commoner in Indians than in other racial groups. Despite improved medical facilities the relative frequency has not fallen much over the years.

Oral cancers have figured prominently in several Chinese biopsy series. Hu and Ch'in (1936) found 69 such cancers in 821 malignancies; Bercovitz (1941), 11 in 451 cancers seen in Hainan. In Peking, 2·5 per cent of 5137 carcinomata in males arose in the buccal cavity, these tumours being sixth in order of relative frequency (Hu and Yang, 1959). In 10,000 malignant tumours in males collected by the Shanghai tumour registry 3·3 per cent were oral, as were 1·5 per cent of the 18,824 cancers in women (Department of Pathology, Shanghai, 1959).

De Leon (1928) reported 315 mouth tumours in 1502 malignant neoplasms seen in the Philippines, while Sta. Cruz and Oca (1946) found that oral cancer ranked third in frequency, only tumours of the breast and uterus being commoner.

A fairly high relative frequency of lingual cancer has been noted in Korea, 5·5 per cent of all carcinomata (Yun, 1949).

Oral cancer is generally a disease of males (Taylor, 1934). When a high incidence is observed in females it is usually possible to single out some environmental factor, such as the use of betel preparations, or iron deficiency anaemia (Ahlbom, 1936). There seems, as Steiner (1954) has already remarked, to be little or no evidence of a genetic factor for human oral cancer.

*Nasopharynx*

The earliest review of nasopharyngeal carcinoma is almost certainly that published in 1901 by Jackson, who searched the indexes of the English, French, German and Italian literature to find 13 examples, adding one of his own. He found the earliest report was that of Durand-Fardel in 1837.

The mean age at death, and at first diagnosis, of cancer of the nasopharynx is held to be somewhat higher in the Occident than in the East. Godtfredsen (1947) found the peak incidence in his Scandinavian series of 454 cases to be in the sixth decade. In a group of English patients with the same disease, the majority were in the fifth or sixth decades (Chakravorty and Ewing, 1957). However, in a series composed largely of American whites, the peak incidence was in the fourth decade in females, and in the fifth in males (Chakravorty and Frazell, 1960).

In a series of 55 necropsies on male Chinese with this disease, performed in Singapore in 1939–41 and 1948–58, the mean age at death was 45·5 $\pm$ 11·0 years.

The youngest necropsied was 14 years old, the oldest 71. One 60-year old Indian male and five Chinese females were autopsied in the same period, the mean age at death of the latter being 41·2 $\pm$ 9·3 years.

The writer has calculated the mean age at death in Teoh's (1957) necropsy series of Hong-Kong Chinese, the mean for males being 41·6 $\pm$ 13·2 years, and for females 40·4 $\pm$ 13·2 years. Ch'in and Szutu (1940) found the average age in a Canton series to be 43 years.

Differences in peak incidence between Western and Oriental populations may be due to population structure differences : only comparison of age-specific death rates will resolve this point.

Although the youth of many of those with nasopharyngeal cancer never fails to appal, the disease has not been seen in Singapore in children below the age of 14 years. Its occurrence has been reported in 4-year-olds in the United States of America (Chakravorty and Ewing, 1957) and Scandinavia (Godtfredsen, 1947), in a 10-year old white American girl (Batory, 1954), and in 12-year old Indian children (Das *et al.*, 1954 ; Sirsat, 1954).

The incidence of cancer of the nasopharynx is usually estimated as being something less than 1·0 per cent of all malignant tumours. Martin and Blady (1940) estimated that in 1936–38 in New York this site accounted for but 0·0017 per cent of all cancer cases. Godtfredsen (1947) suggested a figure of 0·4 per cent for Scandinavia, Molony (1957) 0·3 per cent for Canada. Simmons and Ariel (1949) found that 0·7 per cent of 19,976 admissions with cancer to a Veterans Administration Hospital in Illinois were due to this disease.

In the United States of America, in 1955, some 0·0015 per cent of cancer deaths registered were ascribed to nasopharyngeal tumours (Gordon, Crittenden and Haenzel, 1961), whereas in Singapore, in 1957–59, the comparable figure was 3·5 per cent (Table II). Elsewhere in the East most of the available data are based on biopsy material.

In Marsden's (1958) Malayan series, already cited, 11·5 per cent of cancers in Chinese males, and 6·0 per cent in Chinese females arose in the nasopharynx. Comparable figures for Malaysians and Indians were respectively, 10·6 per cent and 4·2 per cent, and 1·3 per cent and 0·4 per cent. While this relative frequency is probably exaggerated as diagnosis of this particular tumour is nearly always confirmed by biopsy, Marsden (1958) believes that this is one of the five most frequent tumours in both sexes.

The absence of Malaysians in Singapore post mortem material reflects the reluctance of this racial group to permit necropsy (Muir, 1962).

In the Philippines, in a population of unstated racial composition, 3·7 per cent of 2163 malignant tumours were of nasopharyngeal origin (Sta. Cruz and Dos Santos, 1955).

The incidence of these cancers is believed to be low in India. In 1941–51, 42 cases were encountered at the Tata Memorial Hospital, Bombay. In the same period, 980 tonsillar and 2420 lingual cancers were seen (Das *et al.*, 1954 ; Sirsat, 1954). These findings follow the same pattern as those for Indians in Malaya. Similar observations have been made in Japan (Daito, Sakamoto and Hara, 1952), Ceylon (Cooray, 1944), and in Africa (Elmes and Baldwin, 1947 ; Gelfand, 1949), where these tumours are said to be as rare as in the West.

In China the relative frequency in hospital biopsy series varies enormously. In 10,000 malignant tumours in males collected by the Shanghai tumour registry,

6·1 per cent were of nasopharyngeal origin (Department of Pathology, Shanghai, 1959). In Canton, 56·9 per cent of 3010 carcinomata in males, and 17·4 per cent of 4206 carcinomata in females arose at this site (Hu and Yang, 1959). This cancer seems to be much commoner in South China (whence a good part of the Singapore Chinese population originally derived) than in the North (Hu and Yang, 1959). Chinese in Formosa have been shown to have a high frequency of this tumour, 30·4 per cent of malignancies in males, and 5·3 per cent of those in females (Yeh and Cowdry, 1954).

The accurate assessment of the incidence of this tumour in Chinese living without China is of fundamental importance if the cancer is due to an environmental factor. There seems little doubt that this cancer is of high incidence in Chinese in Malaya and Singapore. From the Western hemisphere there are similar reports. Martinez (1940) noted that, in Cuba, 20 of 69 nasopharyngeal cancers were in Chinese, whereas only 64 of 10,317 malignant tumours in all the other regions of the body combined occurred in this race. Martin and Quan (1951), in New York, observed that 37 of 358 nasopharynx cancers were in Chinese, a race which comprised less than 1 per cent of the population at risk. All of these persons were China-born, but some had migrated at an early age. In Caucasians, 5·5 per cent of cancers of the head and neck were in the nasopharynx ; in Chinese, 82·0 per cent. Largely similar observations were made by Chakravorty and Frazell (1960). Smith (1956), studying mortality statistics for Chinese in the United States of America and Hawaii, noted a marked excess for cancer of the pharynx (145–148) in both sexes when compared with the white and non-white populations.

Writing from Surabaja, Indonesia, Djojopranoto and Marchetta (1959) found that, in 1952–54, 7·9 per cent of 855 cancers in Indonesians (a Malaysian people) and 13·9 per cent of 215 cancers in Chinese, were nasopharyngeal. Numerous examples of " cancer of the neck " were also seen in both races, these being in all probability secondaries from the same primary site.

Such high figures for Indonesians, and for Malaysians in Malaya, have been explained on the grounds of intermarriage with, and ethnic affinity to, Chinese. Many believe, as does Marsden (1958), that there must be more than an external carcinogen to explain the racial predilection shown by this cancer. However, the recent discovery by ten Seldam (1962, personal communication) that this cancer is not uncommon in the natives of the highlands of East New Guinea would seem contrary to such a viewpoint, as these peoples have had no contact with Chinese, and are not related to them ethnically.

Before hunting for a nasopharynx cancer gene in the chromosomes of Chinese, it would be as well to study second and third generation Chinese in the United States of America to see whether the attack rate is falling, as it seems most likely that this particular group of Chinese will have departed further from the original environment than any other.

Few since Dobson (1924) have suggested the nature of the possible carcinogen and fewer have made any attempt to support their theory with experimental work. Hou (1960), acting on the assumption that incense smoke may contain a carcinogen, dropped, thrice weekly for 16 months, an oily extract of the tar from such smoke into the right nasal tract of white mice. No cancers were produced. As a control, a 0·5 per cent oily solution of 20-methylcholanthrene was instilled in the same manner. This known carcinogen caused cancers of the nostril, but none of the nasopharynx. It was concluded that the nasopharynx is refractory to this particular stimulus.

Further work in this area is needed.

### SUMMARY AND CONCLUSIONS

The Singapore morbidity (incidence) from tumours of the buccal cavity and pharynx (140–148), relative to that from all cancers (140–205), is twice that of Connecticut and England and Wales.

The Singapore mortality from these tumours, relative to that from all cancer, is at least three times higher than in Australia, England and Wales, Israel, Japan, and the United States of America (white and non-white populations).

Much of this excess is due to nasopharyngeal cancer (146), the Singapore age-standardised death rates for this tumour alone being of the same order as those for *all* cancers of the buccal cavity and pharynx in the countries selected for comparison.

The age and racial distribution of nasopharyngeal cancer is reviewed: the incidence is peculiarly high in Chinese, not only in China, but also those resident in Singapore, Malaya, Indonesia, Cuba, Hawaii, and the United States of America. A relatively high incidence is also found in Malaysians, and possibly in the inhabitants of the Highlands of East New Guinea. The implications of these findings are discussed.

The problem of oral cancer in the East is discussed, and the high incidence in Indians of both sexes living in Malaya, probably due to the chewing of a betel/tobacco mixture, noted.

I am indebted to Prof. K. Shanmugaratnam, and to Dr. H. L. Stewart, Chief, Laboratory of Pathology, National Cancer Institute, Bethesda, Maryland, for kind help and criticism, and for the facilities to write this report, which forms part of a thesis for the degree of Ph.D. (Malaya).

### REFERENCES

AHLBOM, H. E.—(1936) *Brit. med. J.*, ii, 331.
BATORY, K.—(1954) *J. Pediat.*, **45**, 599.
BERCOVITZ, N.—(1941) *Cancer Res.*, **1**, 154.
CHAKRAVORTY, R. A. AND EWING, M. R.—(1957) *Brit. J. Surg.*, **44**, 388.
CHAKRAVORTY, R. C. AND FRAZELL, E. L.—(1960) *Acta Un. int. Cancr.*, **16**, 1333.
CH'IN, K. Y. AND SZUTU, C.—(1940) *Chin. med. J.*, Suppl. III, p. 94.
COORAY, G. H.—(1944) *Indian J. med. Res.*, **32**, 71.
DAITO, T., SAKAMOTO, H. AND HARA, H. J.—(1952) *Arch. Otolaryng., Chicago*, **56**, 45.
DAS, T., TANEJA, G. M., CHADDAH, M. R. AND MINOCHA, D. B.—(1954) *Ann. Otol., etc., St. Louis*, **63**, 890.
DE LEON, W.—(1928) Far Eastern Association of Tropical Medicine. Transactions of the 7th Congress held in British India, December, 1927. Calcutta (Thacker's Press), Vol. I, p. 91.
DEPARTMENT OF PATHOLOGY, SHANGHAI FIRST MEDICAL COLLEGE—(1959) *Chin. J. Path.*, **5**, 77. (Abstracted: (1959) *Chin. med. J.*, **79**, 94.)
DJOJOPRANOTO, M. AND MARCHETTA, F. C.—(1959) *Arch. Otolaryng., Chicago*, **69**, 155.
DOBSON, W. H.—(1924) *Chin. med. J.*, **38**, 786.
ELMES, B. G. T. AND BALDWIN, R. B. T.—(1947) *Ann. trop. Med. Parasit.*, **41**, 321.

GELFAND, M.—(1949) *S. Afr. med. J.*, **23**, 1010.
GODTFREDSEN, E.—(1947) *Proc. roy Soc. Med.*, **40**, 131.
GORDON, T., CRITTENDEN, M. AND HAENZEL, W.—(1961) National Cancer Institute monograph No. 6. Part II. Cancer Mortality Trends in the United States, 1930–1955. Washington (U.S. Department of Health, Education and Welfare), p. 151.
HOFFMAN, F. L.—(1935) *Amer. J. Cancer*, **24**, 661.
HOU, P. C.—(1960) *Rep. Brit. Emp. Cancer Campgn.*, **37**, 592.
HU, C. H. AND CH'IN, C. Y.—(1936) *Chin. med. J.*, Suppl. I, p. 43.
HU CHENG-HSIANG AND YANG CHIEN—(1959) *Ibid.*, **79**, 409.
JACKSON, C.—(1901) *J. Amer. med. Ass.*, **37**, 371.
KHANOLKAR, V. R.—(1945) *Indian J. med. Res.*, **33**, 299.
*Idem* AND SURYABAI, B.—(1945) *Arch. Path.* (*Lab. Med.*), **40**, 351.
LAWLEY, M.—(1956) *Aust. N.Z. J. Surg.*, **25**, 170.
MARSDEN, A. T. H.—(1958) *Brit. J. Cancer*, **12**, 161.
MARTIN, H. AND QUAN, S.—(1951) *Ann. Otol., etc., St. Louis*, **60**, 168.
MARTIN, H. E. AND BLADY, J. V.—(1940) *Arch. Otolaryng., Chicago*, **32**, 692.
MARTINEZ, E.—(1940) *Bol. Liga Cáncer, Habana*, **15**, 276.
MEKIE, D. E. C. AND LAWLEY, M.—(1954) *Arch. Surg., Chicago*, **69**, 841.
*Idem* AND RANSOME, G.—(1950) *Brit. J. Surg.*, **37**, 344.
MOLONY, T. J.—(1957) *Laryngoscope, St. Louis*, **67**, 1297.
MUIR, C. S.—(1959) *Brit. J. Cancer*, **13**, 595.—(1961) *Ibid.*, **15**, 30.—(1962) *Cancer*, **15**, 354.
*Idem* AND KIRK, R.—(1960) *Brit. J. Cancer*, **14**, 597.
ORR, I. M.—(1933) *Lancet*, *ii*, 575.
PAYMASTER, J. C.—(1957) *Brit. J. Surg.*, **44**, 467.
REDDY, D. G. AND RAO, V. K.—(1957) *Indian J. med. Sci.*, **11**, 791.
SANGHVI, L. D., RAO, K. C. M. AND KHANOLKAR, V. R.—(1955) *Brit. med. J.*, i, 1111.
SHANTA, V. AND KRISHNAMURTHI, S.—(1959) *Brit. J. Cancer*, **13**, 381.
SIMMONS, M. W. AND ARIEL, I. M.—(1949) *Surg. Gynec. Obstet.*, **88**, 763.
SIRSAT, M. V.—(1954) *Indian J. med. Sci.*, **8**, 195.
SMITH, R. L.—(1956) *J. nat. Cancer Inst.*, **17**, 667.
STA. CRUZ, J. Z. AND OCA, M. S.—(1946) *J. Phil. Is. med. Ass.*, **22**, 511.
*Idem* AND DOS SANTOS, R.—(1955) *Ibid.*, **31**, 637.
STEINER, P. E.—(1954) 'Cancer: Race and Geography. Some Etiological, Environmental, Ethnological, Epidemiological, and Statistical Aspects in Caucasoids, Mongoloids, Negroids, and Mexicans'. Baltimore (Williams and Wilkins), p. 242.
STOCKS, P.—(1959) *Bull. World Hlth Org.*, **20**, 697.
TAYLOR, G. W.—(1934) *New Engl. J. Med.*, **210**, 1102.
TEOH, T. B.—(1957) *J. Path. Bact.*, **73**, 451.
WORLD HEALTH ORGANIZATION—(1948) Manual of International Statistical Classification of Diseases, Injuries, and Causes of Death; Sixth Revision of International List of Diseases and Causes of Death; adopted 1948. *Bull. World Hlth Org.*, Suppl. I, Vol. 1, p. 43.—(1959) *Epidem. vit. Stat. Rep.*, 12, 181.—(1960) *Ibid.*, **13**, 468.
YEH, S. AND COWDRY, E. V.—(1954) *Cancer*, **7**, 425.
YUN, I. S.—(1949) *Cancer Res.*, **9**, 370.